# Evolution of socioeconomic inequalities in smoking: results from the Portuguese national health interview surveys

**DOI:** 10.1186/s12889-015-1664-y

**Published:** 2015-03-31

**Authors:** Joana Alves, Anton E Kunst, Julian Perelman

**Affiliations:** Escola Nacional de Saúde Pública, Universidade Nova de Lisboa, Avenida Padre Cruz, 1600-560 Lisbon, Portugal; Department of Public Health, Academic Medical Centre, University of Amsterdam, Room J2-207, PO Box 22660, 1100 DD Amsterdam, The Netherlands

**Keywords:** Socioeconomic status, Inequalities, Smoking, Portugal

## Abstract

**Background:**

Southern European countries were traditionally characterized by a higher prevalence of smoking among high socioeconomic groups. Though, recent studies show a reversal of inequalities in Italy and Spain, for example. We investigated whether this evolution also applied to Portugal by describing the evolution of socioeconomic inequalities in smoking between 1987 and 2006.

**Methods:**

We used data from the four Portuguese national health interview surveys (N = 120,140) carried out so far. Socioeconomic status was measured by the educational and income levels of respondents. Socioeconomic inequalities were measured through Odds Ratios (OR), Relative Inequality Indexes (RII), and Concentration Indexes (CI) on being current, ever, and former smoker, adjusting for sex and age. Analyses were performed separately for men and women, and for different birth cohorts.

**Results:**

Among men, smoking was initially more concentrated in high-socioeconomic status individuals (RII = 0.84, 95% Confidence Intervals [95% CI] 0.76-0.93, 1987) but this pattern reversed in the last survey (RII = 1.49, 95% CI 1.34-1.65, 2005/6). Indeed, higher cessation rates were observed among high-socioeconomic groups among all respondents (RII = 0.89, 95% CI 0.84-0.95), coupled with higher initiation rates among the worse-off in younger cohorts (RII = 1.18, 95% CI 1.05-1.31, for youngest generation, 2005/6). Among women, the richer and more educated smoked more in all surveys (RII = 0.21, 95% CI 0.16-0.27, 2005/6), despite being also more likely to quit (RII = 0.41, 95% CI 0.30-0.55). The pattern among women evolved towards a reduction of inequality, which however remained favourable to the worse-off.

**Conclusions:**

Inequalities have been increasingly unfavourable to the worse-off in Portugal, although better-off women are still more likely to smoke. Worrisome inequality trends have been observed among the youngest generations, which call for the rapid implementation of equity-oriented tobacco control policies.

**Electronic supplementary material:**

The online version of this article (doi:10.1186/s12889-015-1664-y) contains supplementary material, which is available to authorized users.

## Background

A large number of studies show that individuals of low socioeconomic status face a higher burden of disease and mortality [1-3]. Behavioural risk factors, tobacco consumption in particular, are more prevalent among individuals from low socioeconomic positions, possibly contributing to these socioeconomic inequalities in health [3,4].

A model of “tobacco epidemics”, developed by Lopez et al. [5], shows that, in a first stage, tobacco becomes socially accepted and a rising male prevalence is observed. In a second phase, male prevalence reaches a peak (around 50 to 80%) and prevalence among women increases rapidly. In this second phase the prevalence may be higher in individuals with higher education. As the health hazards of tobacco become known, the third phase is characterized by a decrease in prevalence among men. This decrease is mainly explained by reduced prevalence among individuals with higher education, who are, for example, more influenced by health promotion policies. Also, female prevalence reaches a peak (around 35 to 45%). In the last phase of the model, smoking prevalence decreases in both sexes. Smoking becomes more common among low-educated individuals while decreasing faster among high-educated ones, who are better equipped to get informed, understand the risks of tobacco consumption, and act accordingly. Recent evidence confirms that individuals with lower education and income smoke more cigarettes a day, experience a higher probability of smoking, and higher initiation and lower cessation rates [6-8]. Inequalities in tobacco consumption seem even to have been widening despite health policies including smoking bans, taxes, and advertising [9].

International comparisons on educational differences based on national health studies showed that in 1987, in southern countries like Portugal and Spain, higher educated women smoked more [10]. The same was true but to a lesser extent among men. Among younger men, Portugal was the country with the lowest educational differences in smoking, from the countries analysed. Subsequent studies for Greece, Italy and Portugal (data from 1998) however still found that women with higher education smoked more, suggesting that Southern European countries might follow a different path of the smoking epidemics [4]. Smoking inequalities favouring the high-SES in the age group of 16 to 24 years old were found in all countries in 1998, except for Portuguese and Greek women [11].

Little evidence has been however produced so far to assess whether Portugal is moving towards the patterns observed in northern European countries. A regional cross-sectional study from 1999–2000 showed that smoking prevalence was higher among white collar women [12]. Although unemployed men smoked more, there were no smoking differences observed among men according to occupational class [12]. Studies combining different periods only estimated the prevalence for the total population, showing e.g. that smoking among men is stabilizing though still increasing among women [13,14].

This study documents the evolution of socioeconomic inequality in smoking behaviour in Portugal from 1987 to 2006, using education and income as socioeconomic factors. We describe how socioeconomic inequalities in prevalence and cessation evolved in time. As Italian analyses showed smoking inequalities to strongly vary according to birth cohort [15], we also analysed trends according to birth cohort to detect evolving patterns across generations. By doing so, we provide insights about the possible path of the smoking epidemic in Portugal. This information is in turn relevant for tailoring policies to the Portuguese context of inequalities in tobacco consumption.

## Methods

We used data from the four National Health Interview Surveys (NHIS) carried out so far in all mainland Portuguese regions (1987, 1995, 1998/99, and 2005/06). NHIS are cross-sectional studies based on representative samples of non-institutionalized population living in Portugal. Data were collected through face-to-face interviews on health status and disease, socio-demographic indicators and lifestyle, among others. The data from the NHIS are collected by the National Institute for Statistics, available on demand for research purposes, and the methods are reported elsewhere [16]. In this study, all individuals aged 25 to 79 years old were included; younger people were excluded to avoid as much as possible individuals who had not completed their education. Also, older people were excluded to reduce the selective mortality bias. The final sample included 120,140 individuals.

We created three dichotomic variables for smoking: “current”, “former” and “ever” smoker. The “current smoker” variable was based on the question “do you currently smoke?” The last three surveys distinguished the answers “daily”, “occasionally” and “non-smoker”. However, the 1987 NHIS only considered two categories, “daily” and “non-smoker”. In order to compare the four samples, the “current smoker” variable has a value one when the person answers “daily smoker” to the above-referred question, and zero otherwise. The “daily smoker” category included a large majority of smokers (89.4% of smokers smoked daily in 1995, 89.1% in 1998–1999 and 90.3% in 2005–2006), so that the loss of information was relatively minor. In regard to the “former smoker” variable, we used the question “Did you ever smoke?”. This question was asked solely to those who did not currently smoke; possible answers were “daily”, “occasionally”, or “never smoked”. The 1987 NHIS asked “Did you ever smoked regularly?”; hence, the “former smoker” variable has a value one for those who answered “yes” in 1987 and those who answered “daily” in subsequent surveys, and zero otherwise. Occasional smokers were included as never smokers. Finally, the “ever smoker” variable values one if the persons reports to be current or former smoker, and zero otherwise. Current smoking is thus given by the percentage of daily smokers in the sample, ever smoking by the percentage of ever smokers (current or former) within the respondents, and smoking cessation by the percentage of former smokers within the ever smokers.

We categorized education into five categories, on the basis of the highest completed diploma, namely no education (zero to three years of education), pre-primary education (four years of education), primary education (nine years of education), secondary education (12 years of education), and tertiary education (more than 12 years of education). The individual income was calculated applying the Organisation for Economic Co-operation and Development (OECD) modified equivalence scale, giving different weights to different family members (1 to the first adult, 0.5 to the second and other family members older than 14 years old, and 0.3 to individuals less than 14 years old) [17]. In all surveys, the upper income category was open ended, e.g., “€2,000 and above”. To estimate the midpoint for the upper income category we followed Parker and Fenwick method [18].

Socioeconomic inequalities in current smoking and smoking cessation were measured using relative inequality indexes (RII), concentration indexes (CI), and odds ratios (OR). Logistic regressions were performed in order to estimate age-adjusted OR of smoking. The dependent variables were current smoking, smoking cessation and ever smoking. The independent variables were age and education, or age and income. Separate analyses were performed for men and women, for each survey, and for different birth cohorts.

For the sake of brevity, we only display results for RII and CI. An additional file shows results for OR analyses [see Additional file 1].

## Results

Descriptive statistics are presented in Table 1. Approximately 53% of the respondents are women, with a mean age of 52 years old, while men have an average age of 51 years old. The percentage of individuals with no education was high in all NHIS but decreased over time (from 22.8% of men and 35.8% of women in the first survey to 11.0% and 18.3% in the last survey, respectively). A small percentage of individuals had tertiary education (8.7% men and 10.8% women in 2005/06) and secondary education (10.7% of men and 9.6% of women in 2005/06), whereas this value increased comparing with the previous surveys. The percentage of men that ever smoked was almost the same across the surveys (57.3% in 1987 to 55.8% in 2005/06) but the percentage of women that ever smoked more than doubled from 1987 (6.3%) to 2005/2006 (13.8%). Among men, the percentage of current smokers within respondents decreased (35.3% to 29.6%) while the percentage of former smokers within ever smokers increased (38.4% to 47.0%) from 1987 to 2005/06. The percentage of women smoking increased from 4.4 to 9.1% between 1987 and 2005/06. Over this 1987-2005/06 period, the percentage of women who stopped smoking within ever smokers increased too (30.2% to 34.0%).

**Table 1:** **Demographic characteristics of respondents according to education, income, and smoking status, by sex and survey year** ^**1**^

	**Men**				**Women**			
	**1987**	**1995**	**1998/99**	**2005/06**	**1987**	**1995**	**1998/99**	**2005/06**
**Educational level**								
Tertiary education	2.9	5.2	6.1	8.7	1.8	5.6	7.2	10.8
Secondary education	12.8	6.3	8.0	10.7	10.6	5.3	6.7	9.6
Primary education	9.1	20.3	23.8	27.7	5.6	15.2	18.2	22.6
Pre-primary education	52.5	51.1	48.8	41.9	46.1	47.3	46.1	38.7
No education	22.8	17.1	13.3	11.0	35.8	26.6	21.7	18.3
**Income level**								
1st quintile (+)	22.1	21.2	21.7	22.1	20.9	19.5	20.5	20.4
2nd quintile	19.7	18.7	20.3	20.1	18.4	17.3	19.0	18.9
3rd quintile	19.8	20.2	19.6	19.4	19.5	20.6	19.6	19.3
4th quintile	20.6	21.5	18.6	15.8	19.6	21.9	17.3	16.4
5th quintile (−)	17.9	18.3	19.9	22.7	21.6	20.9	23.7	25.1
**Smoking status**								
Ever smokers	57.3	53.0	54.6	55.8	6.3	8.6	11.0	13.8
Current smokers	35.3	30.9	31.1	29.6	4.4	6.1	7.9	9.1
Former smokers	38.4	41.6	43.1	47.0	30.2	28.4	28.0	34.0
**Age** ^**2**^	50.3	50.6	50.5	50.6	51.3	51.6	51.7	51.9
**N**	12 113	15 412	15 463	13 426	13 816	17 427	17 476	15 007
***Daily smokers*** ^***3***^	***33.6***	***32.7***	***32.0***	***28.7***	***5.1***	***7.6***	***10.1***	***11.2***

^1^Educational level, income level, and smoking status reported as percentage.

^2^Age reported as mean value for age in years.

^3^Percentage of daily smokers in Portugal aged 15 years or older according to OECD Health Data (2010) [28].

Among men, no significant differences between educational categories in prevalence were observed in the first three surveys (Table 2). However significant differences were observed between 1987 and 2005/06 NHIS, where in 1987 NHIS inequalities favoured the less educated (RII = 0.84) and in 2005/06 NHIS the inequalities favoured the more educated (RII = 1.49). In 1987 and 1998/99 surveys, the inequalities in smoking according to income were not significant. Both RII and CI for income in 2005/06 indicated that smoking is more concentrated among the poorest (RII = 1.15 and CI = −0.04), contrary to the previous surveys. This reversal in inequalities was not observed in women. Women with lower education were less likely to smoke across all surveys (e.g. RII = 0.21 in the last survey). Also, women with lower income were less likely to smoke in all surveys. Income inequalities decreased slightly across surveys, but remained concentrated among the highest-income women (RII = 0.51 in 2005/06 and CI = 0.19).

**Table 2:** **Age-adjusted inequality measures, per sex, smoking status and survey year**

	**1987**		**1995**		**1998/99**		**2005/06**	
**Current smokers - men**								
RII for education	0.84	[0.76;0.93]	0.95	[0.86;1.05]	0.96	[0.88;1.06]	1.49	[1.34;1.65]
RII for income	0.97	[0.90;1.04]	0.87	[0.82;0.93]	0.97	[0.94;1.01]	1.15	[1.07;1.24]
CI for income	0.10		0.09		0.07		−0.04	
**Current smokers - women**								
RII for education	0.01	[0.00;0.01]	0.02	[0.02;0.03]	0.07	[0.06;0.10]	0.21	[0.16;0.27]
RII for income	0.11	[0.08;0.15]	0.24	[0.19;0.30]	0.36	[0.30;0.44]	0.51	[0.42;0.61]
CI for income	0.54		0.38		0.32		0.19	
**Former smokers - men**								
RII for education	0.82	[0.75;0.90]	0.90	[0.84;0.96]	0.97	[0.91;1.03]	0.89	[0.84;0.95]
RII for income	0.85	[0.78;0.93]	0.92	[0.87;0.98]	0.95	[0.93;0.98]	0.88	[0.83;0.92]
CI for income	−0.05		−0.03		−0.03		0.07	
**Former smokers - women**								
RII for education	1.17	[0.78;1.77]	0.78	[0.51;1.17]	0.60	[0.43;0.85]	0.41	[0.30;0.55]
RII for income	0.89	[0.63;1.27]	0.71	[0.53;0.96]	0.62	[0.48;0.82]	0.60	[0.48;0.76]
CI for income	−0.01		0.08		0.11		0.18	
**Ever smokers - men**								
RII for education	0.72	[0.67;0.77]	0.73	[0.69;0.78]	0.77	[0.72;0.81]	1.04	[0.97;1.11]
RII for income	0.92	[0.88;0.97]	0.83	[0.79;0.86]	0.93	[0.91;0.96]	0.93	[0.89;0.98]
CI for income	0.11		0.11		0.08		0.02	
**Ever smokers -women**								
RII for education	0.01	[0.00;0.01]	0.02	[0.01;0.02]	0.05	[0.04;0.06]	0.11	[0.09;0.13]
RII for income	0.10	[0.07;0.13]	0.20	[0.16;0.24]	0.28	[0.24;0.33]	0.36	[0.31;0.41]
CI for income	0.56		0.42		0.36		0.27	

Legend: 95% confidence intervals in parenthesis.

Smoking cessation was at all periods less likely among men without education than among those with tertiary and secondary education (e.g. RII = 0.89, in 2005–2006). Similarly, men with lower income were also less likely to stop smoking in all surveys (in 2005/06 RII = 0.88 and CI = 0.07). Among women there were only significant inequalities in cessation by education level in the last survey. In 2005/06 NHIS, RII was 0.41 for education and 0.60 for income, implying that women with lower education and income quit less.

Regarding ever smoking among men, RII for education was not significant in the 2005/06 survey. In the previous surveys, ever smoking was concentrated in highest levels of education (RII between 0.72 and 0.77). This reversal was not observed for income, as RII was very close to one (RII between 0.92 and 0.93) for all surveys. Ever smoking was also more concentrated in women with higher education and more income in all surveys. However, in the last survey, the magnitude of inequalities for education decreased (RII increased from 0.05 in 1998/99 to 0.11 in 2005/06).

**Figure 1:**
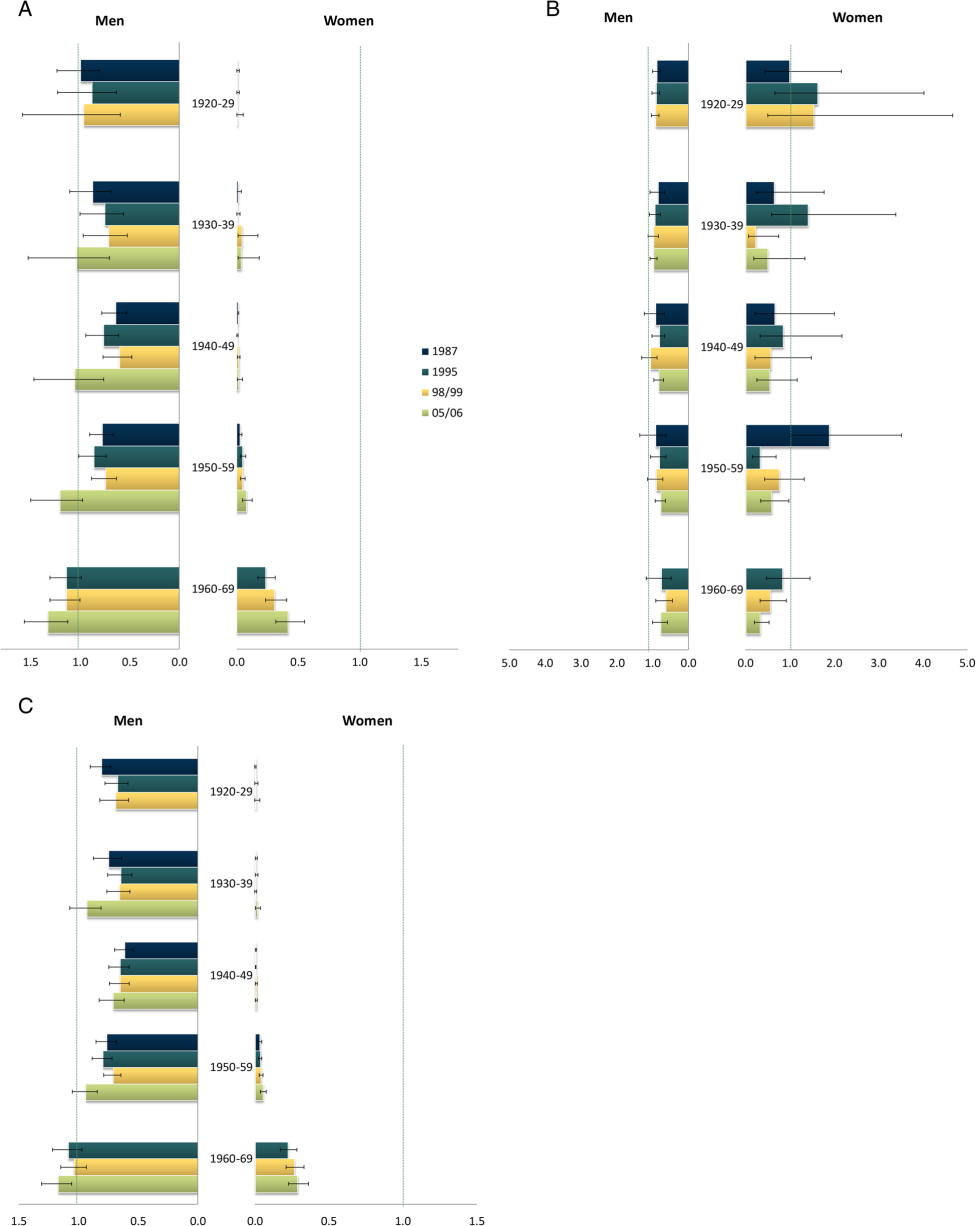
**Education-related relative inequality index for current smokers (A), former smokers (B) and ever smokers (C) by sex, birth cohort and survey year.**

Inequalities in smoking changed mostly in the 1960–69 cohort compared to the previous generations (Figure 1). Education-related inequalities in the last survey for the 1960–1969 generation favoured high-educated men. 1940–1949 and 1950–1959 generations experienced inequalities favouring lower-educated men, except during the 2005/06 survey, when no significant inequalities were observed. Finally, men born in the 1920–1939 period did not experience significant inequalities across education levels in any of the observed years. Across all generations, prevalence was concentrated in women with higher levels of education. However, in the youngest generation the inequalities were less noticeable than in the previous ones. No significant education-related inequalities in cessation were observed by generational cohort, among either men or women. For youngest men generations there were no significant inequalities in ever smoking. In all other generations inequalities favoured the higher-educated men. Women with more education had a higher percentage of ever smokers. Again, the dimension of inequalities was smaller for the youngest cohort.

## Discussion

To the best of our knowledge, this is the first study carried out in Portugal regarding the evolution of smoking inequalities. Findings show that over the 1987–2006 period inequalities in smoking behaviour reversed for men, related to higher cessation rates among high socioeconomic groups in all surveys analysed coupled with higher initiation rates among low socioeconomic groups in the first three surveys, particularly in younger cohorts (1960–1969). A similar trend for current smoking was observed among women but not enough to observe that reversal in the social gradient. This could be explained by higher initiation rates among high socioeconomic groups in all surveys, and the emergence of inequalities in cessation favouring the richer in the last survey, especially in the youngest cohort.

Results confirm that Portugal belongs to the group of Southern countries where women lag behind men in the smoking epidemics [4,5]. However, unlike Italy and Spain, the reversal in inequalities has not been observed yet among younger women [19,20]. For example, in Italy, low educated men aged 25 to 49 years old were more likely to smoke (OR = 1.26), while for women in the same age group inequalities reversed [19]. This suggests either it is too early to observe a reversal, or Portugal experiences a different path.

This last interpretation is consistent with Thun, Peto, Boreham, and Lopez [21]. These authors suggest an update of the epidemiological model of smoking, based on the observation that paths among women significantly vary across countries. For example, in Spain, the late attenuation of smoking cultural prohibition to women delayed women smoking-related mortality and most likely reduced the maximum prevalence levels that would be attained, when compared with countries such as the United States or the United Kingdom [21]. Similarly, a study from Bosdriesz, Mehmedovic, Witvliet, and Kunst found higher prevalence of women smoking in high socioeconomic groups in Latin America and Eastern Mediterranean countries [22]. Authors justify this pattern with the later emancipation of women and with the proximity to Southern European countries, where there is a higher acceptability of smoking among women, coupled with a conservative environment in low socioeconomic and rural groups [22].

Portugal might experience a similar trend with prevalence among women growing late, and with persisting higher prevalence among richer and higher-educated persons. Further study of the most recent trends in countries such as Portugal may show in more detail alternative paths of inequalities in the smoking epidemic.

Although national health surveys are widely used because they provide large sample sizes and important information on health, they suffer from well-known limitations [23]. Firstly, smoking status and cessation were self-reported. However, the validity of self-reports of smoking was showed in most studies [24]. Authors usually recommend validation of smoking status (e.g. by biochemical tests) only in intervention studies, and self-administered questionnaires [24]. Also, the validity of the self-reported smoking status has proved to be high in population-based studies [25]. Self-reported smoking could be a more serious limitation to this study if under-reporting was related to socioeconomic status. For example, in lower socioeconomic classes, characterized by traditional and conservative environments, the acceptability of smoking among women could be lower. If this is the case, our results may over-estimate the pro-rich socioeconomic inequalities in smoking among women.

Secondly, the last survey was from 8 years ago and the inequalities have probably changed by now. In particular, important tobacco policies have been implemented since then, like the protection against involuntary tobacco exposure, implemented by the 2007 legislation. Further study may be relevant to provide evidence on the impact of recent tobacco control policies on inequalities, for which no consistent evidence has been produced yet [26].

## Conclusions

Our results demonstrate an increase of inequalities in cessation, and a reversal of inequalities on smoking among men; thus we may predict a growth of inequalities in health against the worse-off in the future. The trends observed in women also predict the emergence of such inequalities on a near future.

The literature points that tobacco policies have different effects on individuals according to socioeconomic status; for example price increases seem more effective among poorer individuals or those employed in manual occupations, thus reducing inequalities [27]. Our results show a potential widening of inequalities in younger generations; this worrisome trend suggests prioritizing equity-oriented tobacco control strategies such as price increases.

## Additional file

Additional file 1:
**Age-adjusted odds ratio on the probability of being current smoker, ever smoker and former smoker in Portugal, per survey year.**

## Abbreviations

OR: Odds ratio
RII: Relative inequality Index
CI: Concentration Index
95% CI: 95% Confidence Intervals
OECD: Organisation for Economic Co-operation and Development
