# Supplementary material for: Evolution of socioeconomic inequalities in smoking: results from the Portuguese national health interview surveys
Source: BMC Public Health. 2015 Mar 31;15:311. doi: 10.1186/s12889-015-1664-y (PMC4391133; doi:10.1186/s12889-015-1664-y)
Supplement: Additional file 1: — Age-adjusted odds ratio on the probability of being current smoker, ever smoker and former smoker in Portugal, per survey year. [file 12889_2015_1664_MOESM1_ESM.docx]

### Table 3. Age-adjusted OR on the probability of being current smoker in Portugal, per survey year (N=120,140).

| **Current smokers** | **1987** | | **1995** | | **1998/99** | | **2005/06** | |
| --- | --- | --- | --- | --- | --- | --- | --- | --- |
| **Education - men** |  |  |  |  |  |  |  |  |
| Tertiary and second. | 1.00 |  | 1.00 |  | 1.00 |  | 1.00 |  |
| Primary education | 1.08 | [0.93;1.26] | 1.36 | [1.20;1.54] | 1.42 | [1.27;1.59] | 1.58 | [1.41;1.76] |
| Pre-primary educ. | 0.73 | [0.65;0.81] | 1.08 | [0.97;1.22] | 1.10 | [0.99;1.23] | 1.58 | [1.41;1.77] |
| No education | 0.89 | [0.77;1.02] | 1.05 | [0.90;1.22] | 1.01 | [0.86;1.18] | 1.54 | [1.30;1.82] |
| **Income - men** |  |  |  |  |  |  |  |  |
| 1st quintile (+) | 1.00 |  | 1.00 |  | 1.00 |  | 1.00 |  |
| 2nd quintile | 0.96 | [0.85;1.08] | 1.03 | [0.93;1.15] | 0.99 | [0.89;1.11] | 1.32 | [1.17;1.48] |
| 3rd quintile | 0.88 | [0.78;1.00] | 1.00 | [0.89;1.11] | 0.97 | [0.87;1.09] | 1.27 | [1.12;1.43] |
| 4th quintile | 0.86 | [0.76;0.98] | 1.01 | [0.91;1.13] | 0.97 | [0.86;1.09] | 1.42 | [1.25;1.61] |
| 5th quintile (-) | 0.87 | [0.76;0.99] | 0.83 | [0.73;0.93] | 0.92 | [0.82;1.04] | 1.40 | [1.24;1.58] |
| **Education - women** |  |  |  |  |  |  |  |  |
| Tertiary and second. | 1.00 |  | 1.00 |  | 1.00 |  | 1.00 |  |
| Primary education | 0.54 | [0.42;0.69] | 0.60 | [0.52;0.70] | 0.72 | [0.63;0.83] | 0.96 | [0.84;1.09] |
| Pre-primary educ. | 0.09 | [0.08;0.12] | 0.15 | [0.13;0.18] | 0.21 | [0.18;0.25] | 0.38 | [0.32;0.45] |
| No education | 0.04 | [0.02;0.06] | 0.04 | [0.03;0.07] | 0.12 | [0.08;0.17] | 0.12 | [0.08;0.19] |
| **Income - women** |  |  |  |  |  |  |  |  |
| 1st quintile (+) | 1.00 |  | 1.00 |  | 1.00 |  | 1.00 |  |
| 2nd quintile | 0.36 | [0.29;0.45] | 0.43 | [0.36;0.52] | 0.66 | [0.56;0.77] | 0.65 | [0.55;0.77] |
| 3rd quintile | 0.18 | [0.13;0.24] | 0.41 | [0.34;0.50] | 0.46 | [0.39;0.54] | 0.64 | [0.54;0.76] |
| 4th quintile | 0.17 | [0.12;0.24] | 0.28 | [0.23;0.35] | 0.40 | [0.33;0.49] | 0.56 | [0.47;0.68] |
| 5th quintile (-) | 0.11 | [0.08;0.17] | 0.26 | [0.21;0.33] | 0.33 | [0.27;0.41] | 0.49 | [0.41;0.59] |

Legend: 95% confidence intervals in parenthesis.

### Table 4. Age-adjusted OR on the probability of being former smoker in Portugal, per survey year (N=120,140).

| **Former smokers** | **1987** | | **1995** | | **1998/99** | | **2005/06** | |
| --- | --- | --- | --- | --- | --- | --- | --- | --- |
| **Education - men** |  |  |  |  |  |  |  |  |
| Tertiary and second. | 1.00 |  | 1.00 |  | 1.00 |  | 1.00 |  |
| Primary education | 0.91 | [0.74;1.13] | 0.77 | [0.65;0.92] | 0.85 | [0.72;0.99] | 0.83 | [0.72;0.97] |
| Pre-primary educ. | 0.99 | [0.86;1.15] | 0.79 | [0.68;0.93] | 0.90 | [0.77;1.04] | 0.75 | [0.65;0.87] |
| No education | 0.69 | [0.58;0.83] | 0.59 | [0.48;0.72] | 0.68 | [0.55;0.83] | 0.55 | [0.45;0.68] |
| **Income - men** |  |  |  |  |  |  |  |  |
| 1st quintile (+) | 1.00 |  | 1.00 |  | 1.00 |  | 1.00 |  |
| 2nd quintile | 0.89 | [0.76;1.05] | 0.89 | [0.77;1.03] | 0.92 | [0.79;1.07] | 0.73 | [0.63;0.85] |
| 3rd quintile | 0.92 | [0.78;1.08] | 0.92 | [0.79;1.06] | 0.85 | [0.73;0.99] | 0.73 | [0.63;0.86] |
| 4th quintile | 0.84 | [0.71;0.99] | 0.78 | [0.67;0.91] | 0.82 | [0.70;0.96] | 0.64 | [0.54;0.75] |
| 5th quintile (-) | 0.74 | [0.62;0.88] | 0.75 | [0.64;0.89] | 0.82 | [0.71;0.96] | 0.59 | [0.50;0.68] |
| **Education - women** |  |  |  |  |  |  |  |  |
| Tertiary and second. | 1.00 |  | 1.00 |  | 1.00 |  | 1.00 |  |
| Primary education | 0.69 | [0.43;1.11] | 0.87 | [0.67;1.13] | 0.99 | [0.79;1.25] | 0.71 | [0.58;0.87] |
| Pre-primary educ. | 1.47 | [1.03;2.09] | 0.70 | [0.51;0.96] | 0.71 | [0.53;0.95] | 0.49 | [0.37;0.64] |
| No education | 0.85 | [0.38;1.93] | 1.18 | [0.56;2.46] | 0.64 | [0.34;1.22] | 0.43 | [0.21;0.88] |
| **Income - women** |  |  |  |  |  |  |  |  |
| 1st quintile (+) | 1.00 |  | 1.00 |  | 1.00 |  | 1.00 |  |
| 2nd quintile | 0.83 | [0.56;1.25] | 0.94 | [0.69;1.29] | 0.63 | [0.48;0.84] | 0.87 | [0.67;1.12] |
| 3rd quintile | 1.34 | [0.82;2.19] | 0.65 | [0.45;0.93] | 0.75 | [0.56;1.02] | 0.67 | [0.50;0.88] |
| 4th quintile | 0.86 | [0.45;1.65] | 0.78 | [0.53;1.15] | 0.60 | [0.41;0.88] | 0.64 | [0.47;0.87] |
| 5th quintile (-) | 1.03 | [0.49;2.15] | 0.74 | [0.47;1.17] | 0.70 | [0.48;1.03] | 0.50 | [0.36;0.69] |

Legend: 95% confidence intervals in parenthesis.

### Table 5. Age-adjusted OR on the probability of being ever smoker in Portugal, per survey year (N=120,140).

| **Ever smokers** | **1987** | | **1995** | | **1998/99** | | **2005/06** | |
| --- | --- | --- | --- | --- | --- | --- | --- | --- |
| **Education - men** |  |  |  |  |  |  |  |  |
| Tertiary and second. | 1.00 |  | 1.00 |  | 1.00 |  | 1.00 |  |
| Primary education | 1.02 | [0.87;1.20] | 1.19 | [1.05;1.34] | 1.35 | [1.21;1.50] | 1.45 | [1.31;1.60] |
| Pre-primary educ. | 0.57 | [0.51;0.64] | 0.79 | [0.71;0.88] | 0.87 | [0.79;0.96] | 1.22 | [1.11;1.35] |
| No education | 0.58 | [0.50;0.66] | 0.65 | [0.57;0.74] | 0.69 | [0.61;0.79] | 1.05 | [0.91;1.21] |
| **Income - men** |  |  |  |  |  |  |  |  |
| 1st quintile (+) | 1.00 |  | 1.00 |  | 1.00 |  | 1.00 |  |
| 2nd quintile | 0.85 | [0.75;0.96] | 0.91 | [0.83;1.01] | 0.88 | [0.79;0.97] | 1.08 | [0.97;1.20] |
| 3rd quintile | 0.78 | [0.69;0.88] | 0.90 | [0.81;0.99] | 0.79 | [0.71;0.88] | 1.00 | [0.90;1.11] |
| 4th quintile | 0.71 | [0.63;0.80] | 0.82 | [0.74;0.90] | 0.79 | [0.71;0.88] | 1.03 | [0.92;1.16] |
| 5th quintile (-) | 0.62 | [0.55;0.71] | 0.60 | [0.54;0.66] | 0.71 | [0.64;0.79] | 0.92 | [0.83;1.02] |
| **Education - women** |  |  |  |  |  |  |  |  |
| Tertiary and second. | 1.00 |  | 1.00 |  | 1.00 |  | 1.00 |  |
| Primary education | 0.46 | [0.37;0.58] | 0.54 | [0.47;0.61] | 0.68 | [0.60;0.76] | 0.77 | [0.69;0.86] |
| Pre-primary educ. | 0.09 | [0.08;0.11] | 0.12 | [0.10;0.14] | 0.15 | [0.13;0.18] | 0.23 | [0.20;0.27] |
| No education | 0.03 | [0.02;0.04] | 0.04 | [0.03;0.06] | 0.08 | [0.06;0.10] | 0.07 | [0.05;0.09] |
| **Income - women** |  |  |  |  |  |  |  |  |
| 1st quintile (+) | 1.00 |  | 1.00 |  | 1.00 |  | 1.00 |  |
| 2nd quintile | 0.32 | [0.26;0.39] | 0.39 | [0.33;0.45] | 0.52 | [0.45;0.60] | 0.55 | [0.47;0.63] |
| 3rd quintile | 0.18 | [0.14;0.23] | 0.33 | [0.28;0.39] | 0.37 | [0.32;0.43] | 0.48 | [0.41;0.55] |
| 4th quintile | 0.15 | [0.11;0.20] | 0.23 | [0.19;0.28] | 0.30 | [0.25;0.36] | 0.41 | [0.35;0.48] |
| 5th quintile (-) | 0.10 | [0.07;0.15] | 0.22 | [0.18;0.27] | 0.26 | [0.22;0.31] | 0.33 | [0.28;0.38] |

Legend: 95% confidence intervals in parenthesis.
